# Eleven nominal species of *Burmoniscus* are junior synonyms of *B. kathmandius* (Schmalfuss, 1983) (Crustacea, Isopoda, Oniscidea)

**DOI:** 10.3897/zookeys.607.8253

**Published:** 2016-07-25

**Authors:** Shigenori Karasawa

**Affiliations:** 1 Faculty of Education, Fukuoka University of Education, 1-1 Akamabunkyo-machi, Munakata, Fukuoka, 811-4192 Japan; 2 Faculty of Regional Sciences, Tottori University, 4-101 Koyama-machi Minami, Tottori, 680-8551 Japan (Present)

**Keywords:** COI, 12S rRNA, 16S rRNA, Japan, Philosciidae, taxonomy

## Abstract

Holotypes, paratypes, and specimens newly collected from the type localities (i.e., topotypes) of *Burmoniscus aokii* (Nunomura, 1986), *Burmoniscus boninensis* (Nunomura, 1986), *Burmoniscus daitoensis* (Nunomura, 1986), *Burmoniscus hachijoensis* Nunomura, 2007, *Burmoniscus japonicus* (Nunomura, 1986), *Burmoniscus kagoshimaensis* Nunomura, 2003, *Burmoniscus murotoensis* (Nunomura, 1986), *Burmoniscus okinawaensis* (Nunomura, 1986), *Burmoniscus shibatai* (Nunomura, 1986), *Burmoniscus tanabensis* Nunomura, 2003, and *Burmoniscus watanabei* (Nunomura, 1986) were examined in order to clarify their taxonomic status. Observation of 13 morphological characters that were purposed to show species-level diagnostic variations in the original descriptions suggests that all eleven nominal species are identical, and molecular analysis based on three gene fragments supports this suggestion. Additionally, the morphology of the carpus of pereopod 1 and of the endo- and exopodites of pleopod 1 of these species are consistent with those of *Burmoniscus kathmandius* (Schmalfuss, 1983). The eleven above-mentioned species of *Burmoniscus* described from Japan are therefore relegated to junior synonyms of *Burmoniscus kathmandius*, originally reported from Nepal.

## Introduction

The genus *Burmoniscus* Collinge, 1914 includes more than 100 nominal species, 14 of which have been recorded in Japan (Nunomura 2003a,b, 2007, Schmalfuss 2004, Karasawa and Honda 2012, Karasawa and Goto 2014), but the taxonomy of this genus remains poorly understood (Karasawa and Honda 2012, Karasawa and Goto 2014). In Japan, these species were originally described as belonging to the genus *Setaphora* Budde-Lund, 1909 (Nunomura 1986), which has been shown to be a synonym of *Anchiphiloscia* Stebbing, 1908 (Ferrara and Taiti 1986). Subsequently, Taiti and Ferrara (1991) assigned *Setaphora okinawaensis* Nunomura, 1986, to *Burmoniscus* based on examination of specimens from Hawaii. Additionally, these authors suspected that several of the nominal species from Japan that had been described by Nunomura (1986) were identical to *Burmoniscus okinawaensis*. In 1993, Kwon and Jeon (1993) re-examined type specimens of *Setaphora aokii* Nunomura, 1986, *Setaphora boninensis* Nunomura, 1986, *Setaphora daitoensis* Nunomura, 1986, *Setaphora japonica* Nunomura, 1986, *Setaphora murotoensis* Nunomura, 1986, *Setaphora shibatai* Nunomura, 1986, *Setaphora watanabei* Nunomura, 1986, and *Burmoniscus okinawaensis*, and concluded that all these species belonged to *Burmoniscus* and were identical. The authors also proposed *Burmoniscus okinawaensis* as the valid name. More recent studies, however, have suggested that these species are eight valid species of this genus in Japan (e.g., Schmalfuss 2004, Nunomura 2011, 2015). The three most recently described congeners from Japan, viz., *Burmoniscus kagoshimaensis* Nunomura, 2003, *Burmoniscus tanabensis* Nunomura, 2003, and *Burmoniscus hachijoensis* Nunomura, 2007, might be ascribed to the *Burmoniscus okinawaensis* complex. Schmalfuss (1983) initially described a species collected from Nepal as *Rennelloscia kathmandia* Schmalfuss, 1983 but subsequently moved it to *Burmonicus* (Schmalfuss 2004). Not appreciating that *Burmoniscus* is masculine in gender, Karasawa et al. (2012) mistakenly referred to this species as *Burmoniscus kathmandia* whereas the correct spelling is *Burmoniscus kathmandius*. The morphological characteristics of this species, including the two convex regions of the tip of the pleopod 1 endopodite in males, the branched setae of the carpus, and the shape of the male’s pleopod 1 exopodite, are consistent with those of *Burmoniscus okinawaensis* described by Nunomura (1986) and Taiti and Ferrara (1991). Thus, I suspected that eleven of the 14 nominal species of *Burmoniscus* in Japan are not only identical to each other, but are in fact junior synonyms of *Burmoniscus kathmandius*.

The objective of the present study was to redescribe the purportedly diagnostic morphological features of the type specimens, or of new material collected from the type localities (topotypes), of these eleven *Burmoniscus* species from Japan, and thus determine whether or not they are distinct from *Burmoniscus kathmandius*.

## Material and methods

### Sample collection

**Figure 1.**
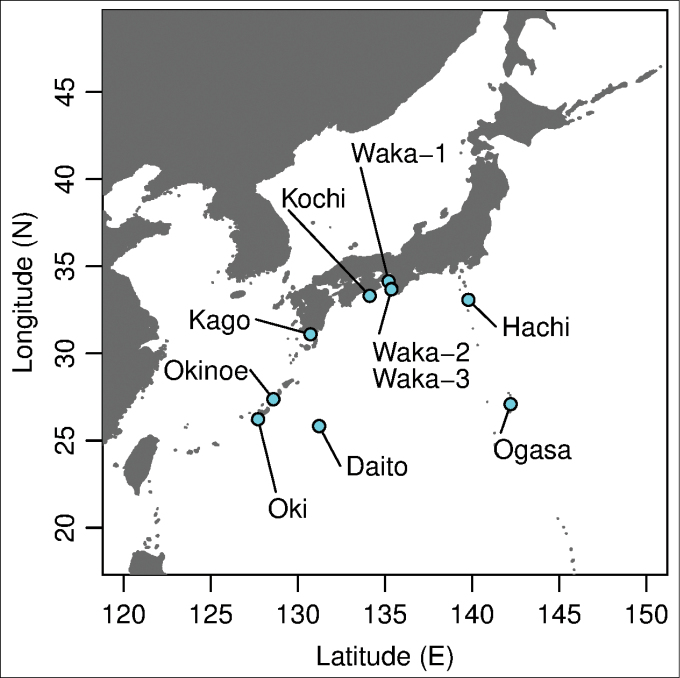
Map of type localities of the 11 *Burmoniscus* species. Daito: Minami-daitojima Island, Okinawa; Hachi: Hachijojima Island, Tokyo; Kago: Sata Town, Kagoshima; Kochi: Muroto City, Kochi; Ogasa: Chichijima Island, Ogasawara Islands, Tokyo; Oki: Naha City, Okinawa; Okinoe: Okinoerabujima Island, Kagoshima; Waka-1: Kainan City, Wakayama; Waka-2: Tanabe City, Wakayama; Waka-3: Shirahama Town, Wakayama.

Holotypes or paratypes were examined when possible; however, when such specimens were in poor condition or required dissection, new specimens collected from the type localities (topotypes) were examined instead. I was unable to collect specimens of *Burmoniscus aokii* and *Burmoniscus boninensis* from their type localities on Chichijima Island, so new specimens from another site on Chichijima Island were examined instead. In addition, because efforts to collect new specimens of *Burmoniscus hachijoensis* from the type locality on Hachijojima Island failed, some specimens were collected from another site on this island. The type localities of the eleven species of *Burmoniscus* are illustrated in Figure 1 and detailed collection data are provided in Suppl. material 1. For the sake of clarity, the current report tentatively treats all the topotypic (or near-topotypic) material as the respective nominal species described by Nunomura (1986, 2003a,b, 2007). Voucher specimens are deposited in the collection of the Kitakyushu Museum of Natural History and Human History (KMNH-IvR), Kitakyushu, Fukuoka Prefecture, Japan.

### Morphology

Male specimens were used for morphological examination, except for pereopod 1 of *Burmoniscus aokii* and *Burmoniscus boninensis* and maxilla 1 of *Burmoniscus tanabensis*, of which only female had these appendages unbroken. The antenna 1, maxilliped, genital papilla, endopodites and exopodites of pleopods 1 and 2, and pereonites 1–7 were unilaterally removed from the body of each specimen under a stereo microscope (SZH, Olympus Corp., Japan). These body parts were then placed in Hoyer’s mounting medium (Krantz and Walter 2009) on slides, gently covered with a coverslip, and drawn under a microscope (Eclipse E400, NIKON Corp., Japan). The b/c and d/c co-ordinates of the *noduli laterales* were calculated following the method of Vandel (1962). The epimeron 7 and pleotelson were drawn using a stereo microscope (SZH) or a digital microscope (VHX-2000, KEYENCE Corp., Japan). Scanning electron microscopy (SEM) was used to visualize the morphology of the ommatidia, the outer endite of maxilla 1, and pereopod 1. These three parts were removed from the body, dried at room temperature, then placed on aluminum stubs and coated with gold. SEM photos were taken using a JCM-5100 (JEOL Ltd., Japan). Exopodite length of the uropod and head width were measured using a digital microscope, and the length of the uropod was standardized by calculating the ratio of exopodite length to head width to avoid confounding effects of body size. The voucher specimens used for morphological analysis are listed in Suppl. material 2.

### Molecular analysis

A single topotypic (or near-topotypic) material of the eleven species was used for molecular analysis, but a single specimen for *Burmoniscus aokii* and *Burmoniscus boninensis* was used. Total DNA was extracted from leg muscle using the Qiagen DNeasy Blood and Tissue Kit, according to the manufacturer’s protocol (Qiagen, Germany). Parts of the mitochondrial cytochrome c oxidase subunit I (COI) and mitochondrial 12S and 16S ribosomal RNA (rRNA) genes were amplified by polymerase chain reaction (PCR) using the following primers: LCO1490 and HCO2198 (Folmer et al. 1994) for the COI region, 12Sai and 12Sbi (Palumbi 1996) for the 12S rRNA region, and 16Sar and 16Sbr (Klossa-Kilia et al. 2006) for the 16S rRNA region. If the 16S rRNA region could not be amplified using these primers, a different primer, 16Sar-int-sf (Parmakelis et al. 2008), was used instead. PCR was carried out in 20-µl reaction volumes with Ex Taq (Takara Bio, Japan). The cycle program comprised an initial denaturation step at 94°C for 3 min followed by 30 cycles of 1 min at 94°C, 1 min at 44–48°C, and 1 min at 72°C, and finally a 7-min extension at 72°C. PCR products were purified using a illustra ExoProStar (GE Healthcare Japan Corp., Japan) and directly sequenced by Macrogen Japan (Japan) using the same primer sets used for PCR. *Burmoniscus* sp., *Burmoniscus meeusei* (Holthuis, 1947), *Burmoniscus dasystylus* Nunomura, 2003, *Burmoniscus ocellatus* (Verhoeff, 1928), and *Ligidium ryukyuensis* Nunomura, 1983 from Japan were also added to the molecular analysis, the last as an outgroup. No material *Burmoniscus kathmandius* from Nepal was available. Sample details and accession numbers are given in Suppl. material 1.

The sequences were aligned using the default settings in MUSCLE 3.5 (Edgar 2004) at SeaView 4 (Gouy et al. 2010). Gaps were excluded from subsequent analyses. Maximum Likelihood (ML) analysis was performed using RAxML Version 8 (Stamatakis 2014). The best-fit models of sequence evolution for both gene and codon, as determined by the Akaike Information Criterion correction (AICc) in the program KAKUSAN 4 (Tanabe 2011), were partitioned equal-mean-rate models. Bootstrap support was assessed using 1000 replicates. Genetic distances were calculated as p-distances using MEGA 6 (Tamura et al. 2013).

## Results and discussion

**Figure 2.**
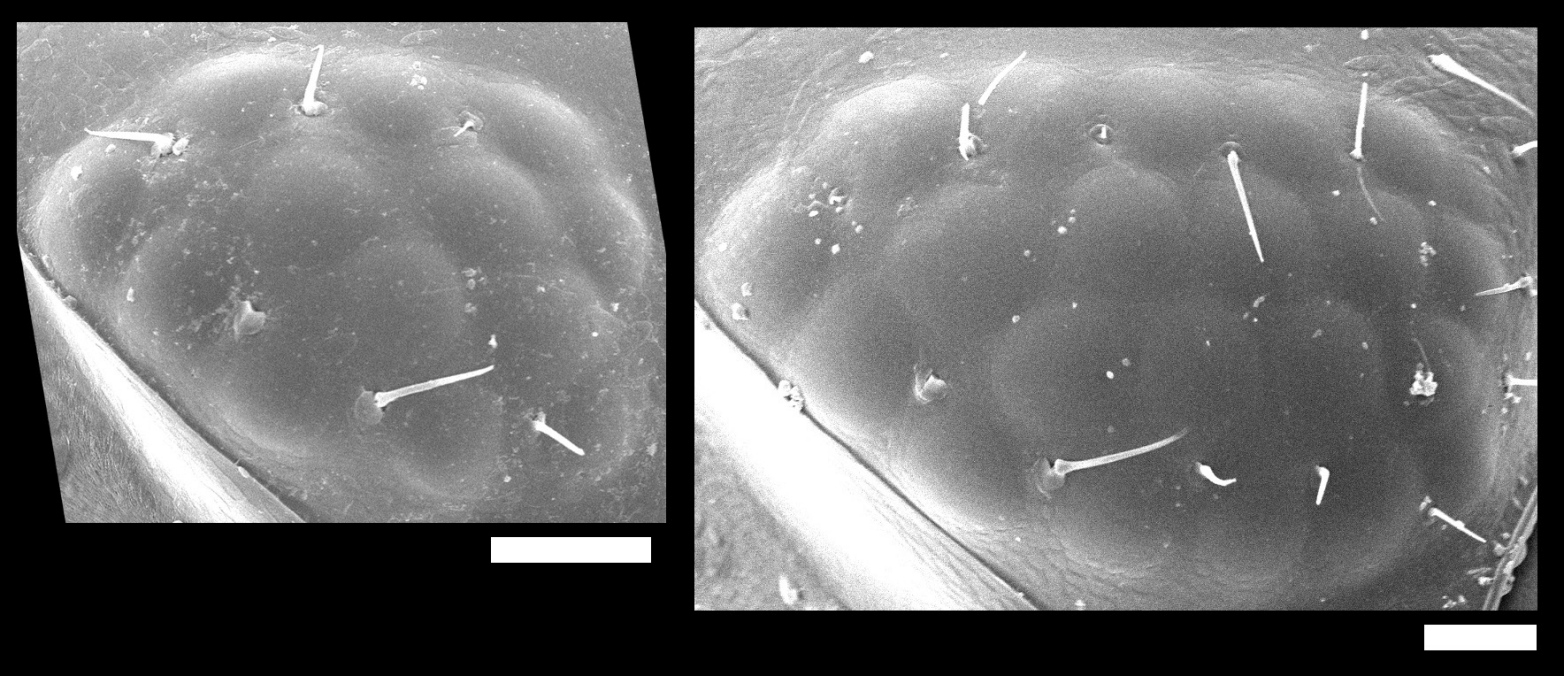
SEM photos of ommatidia of male specimens collected from type locality of *Burmoniscus okinawaensis*, KMNH-IvR-500828 and -500829. Scale bars: 50 µm.

**Eye.** The number of ommatidia varied considerably among the nominal species. *Burmoniscus hachijoensis* had the fewest ommatidia (12), but most species had more than 20 ommatidia (Suppl. material 3). Nunomura (2003a) argued that *Burmoniscus tanabensis* could be distinguished from *Burmoniscus watanabei* and *Burmoniscus okinawaensis* because it has fewer ommatidia. In the present study, however, the number of ommatidia varied from 13 to 21 within a population from the type locality of *Burmoniscus okinawanesis* (Fig. 2), and these numbers might be somewhat correlated with body size. Thus, the number of ommatidia is not a reliable feature for separating these species of *Burmoniscus*.

**Figure 3.**
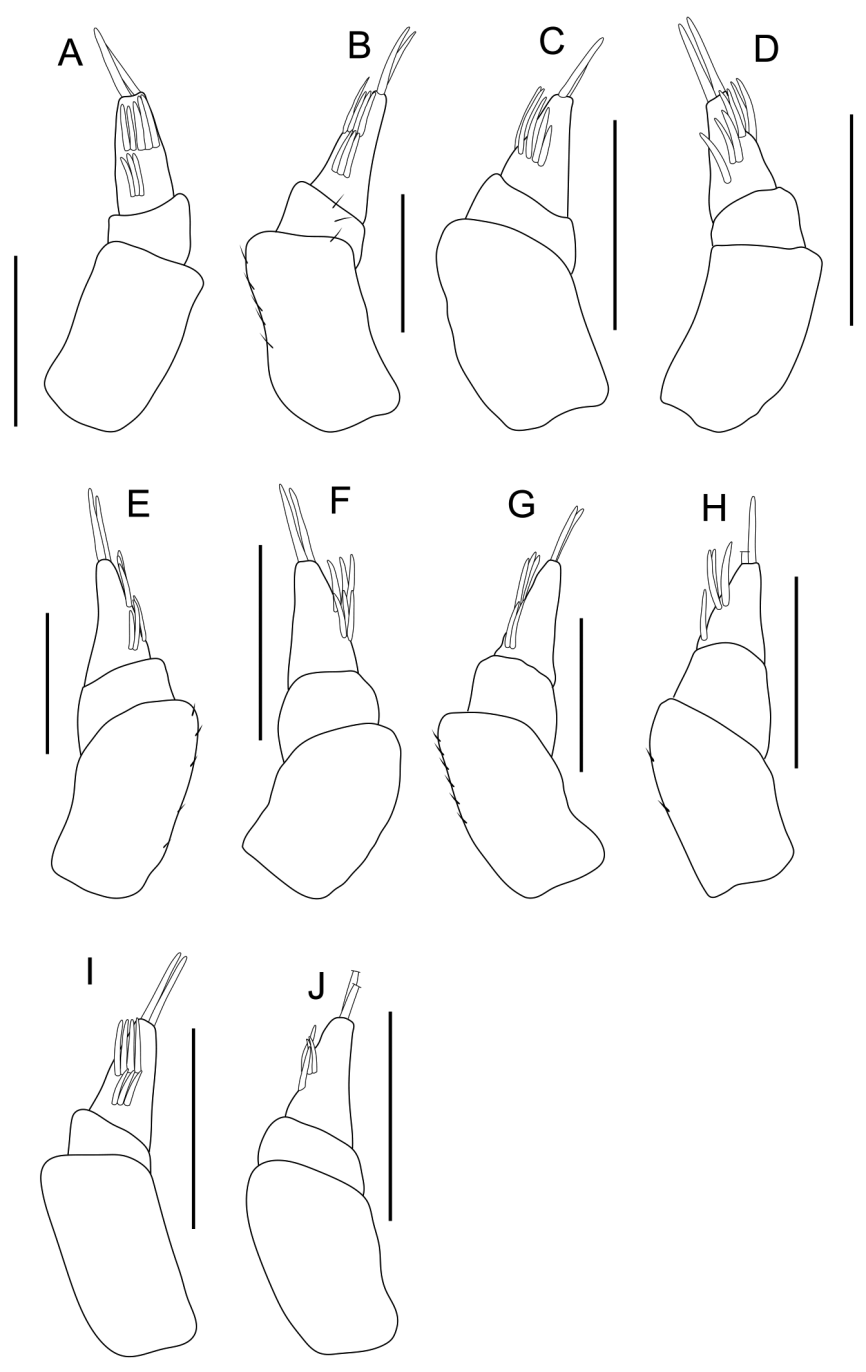
Antenna 1. **A** specimen collected from Chichijima Island (including type localities of *Burmoniscus aokii* and *Burmoniscus boninensis*), KMNH-IvR-500809 **B** *Burmoniscus daitoensis*, KMNH-IvR-500811 **C** *Burmoniscus hachijoensis*, KMNH-IvR-500814 **D** *Burmoniscus japonicus*, KMNH-IvR-500817 **E** *Burmoniscus kagoshimaensis*, KMNH-IvR-500821 **F** *Burmoniscus murotoensis*, KMNH-IvR-500824 **G** *Burmoniscus okinawaensis*, KMNH-IvR-500830 **H** *Burmoniscus shibatai*, KMNH-IvR-500834 **I** *Burmoniscus tanabensis*, KMNH-IvR-500837 **J** *Burmoniscus watanabei*, KMNH-IvR-500842. All specimens male. Scale bars: 100 µm.

**Antenna 1.** As re-described from newly collected topotypes (Fig. 3), the antennae 1 of all specimens consisted of three articles and the apical article bore numerous aesthetascs. Two of these were long and located at the tip, while the others were short and located on the lateral margin. There was variation in the number of short aesthetascs among specimens (4–8). As in the original descriptions of *Burmoniscus* species, the total number of aesthetascs ranged from 2 to 11 (Suppl. material 3). The number of aesthetascs was used to distinguish *Burmoniscus tanabensis* and *Burmoniscus hachijoensis* by Nunomura (2003a, 2007), as both species had more aesthetascs than *Burmoniscus okinawaensis* and *Burmoniscus watanabei*. However, there has been no comparison of *Burmoniscus tanabensis* and *Burmoniscus hachijoensis* to other species with more than five aesthetascs. For example, Nunomura (1986) described the antenna 1 of *Burmoniscus shibatai* as having a total of eleven aesthetascs, but this species was not discussed by him later (Nunomura 2003a, 2007). Moreover, the original descriptions of some species mentioned only two aesthetascs on antenna 1, but small aesthetascs were always present in addition to these, even if they were minute and difficult to observe. It is possible that their presence was overlooked in the original descriptions. Taken together, these observations suggest that the number of aesthetascs on antenna 1 is not suitable for distinguishing among species of *Burmoniscus*.

**Figure 4.**
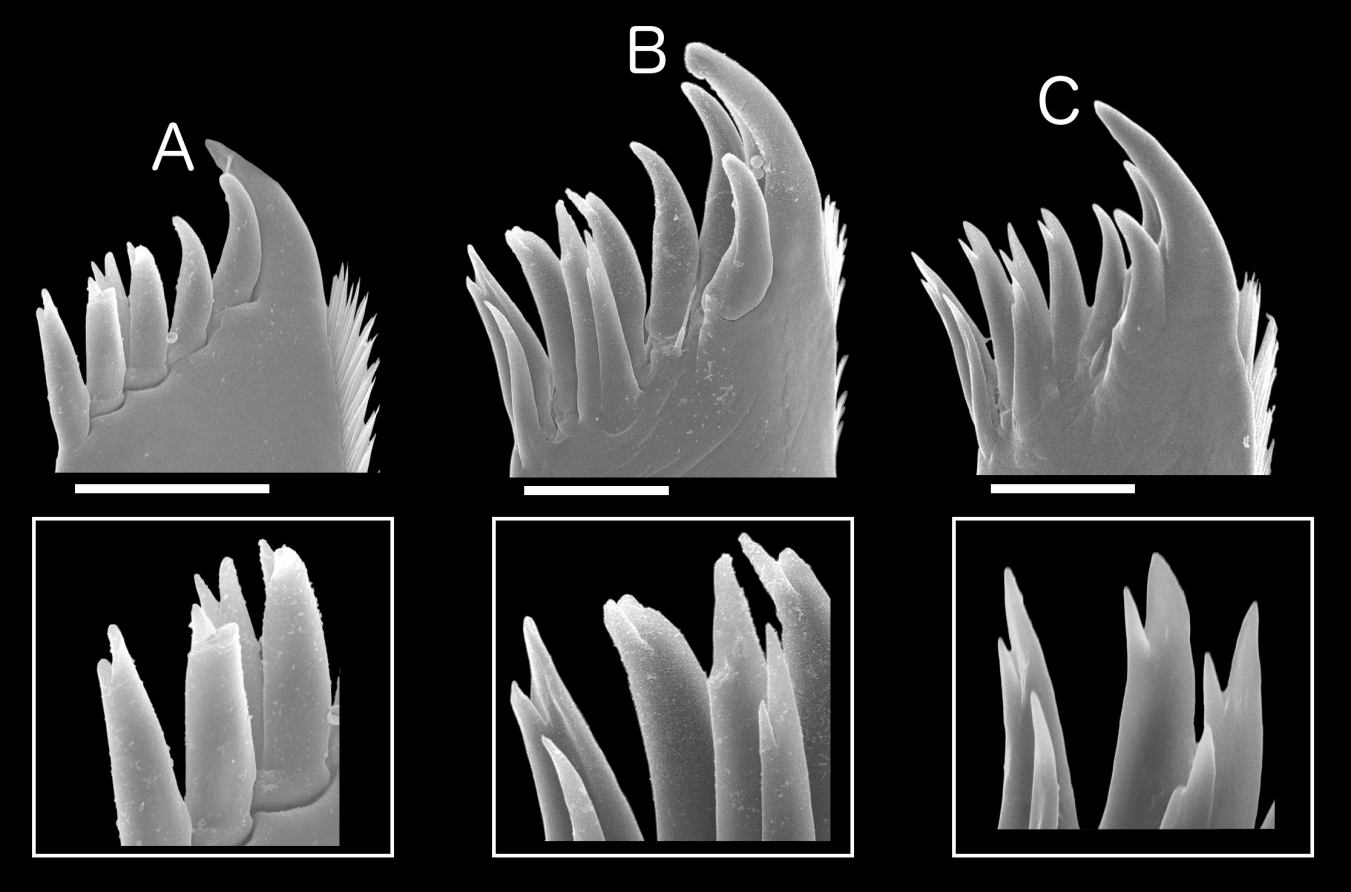
SEM photos of outer endite of maxilla 1 of specimens collected from the type localities of *Burmoniscus tanabensis* (**A** male and **B** female) and *Burmoniscus okinawaensis* (**C** male), with details of setal tips. **A** KMNH-IvR-500838 **B** KMNH-IvR-500839 **C** KMNH-IvR-500830. Scale bars: 50 µm.

**Outer endite of maxilla 1.** The outer endites of maxillae 1 of the eleven nominal species of *Burmoniscus* with which we are concerned all bore 10 setae, both simple and bifid. However, there was variation in the number of simple and bifid types among species. For example, *Burmoniscus shibatai* and *Burmoniscus tanabensis* had only simple setae, while other species had 2–6 bifid setae (Suppl. material 3). Nunomura (2003a) used the lack of bifid setae on maxilla 1 as a taxonomic characteristic distinguishing *Burmoniscus tanabensis* from *Burmoniscus okinawaensis* and *Burmoniscus watanabei*. However, a single bifid seta is present in the figure accompanying the original description of *Burmoniscus tanabensis* (fig. 3G in Nunomura 2003a). Examination of new topotypic specimens of *Burmoniscus tanabensis* and *Burmoniscus okinawaensis* showed that *Burmoniscus tanabensis* bears several bifid setae on maxilla 1 (Fig. 4). This suggests that the numbers of the two types of setae cannot be used to distinguish *Burmoniscus tanabensis*. Whether such variation is useful to distinguish among other *Burmoniscus* species remains unknown.

**Figure 5.**
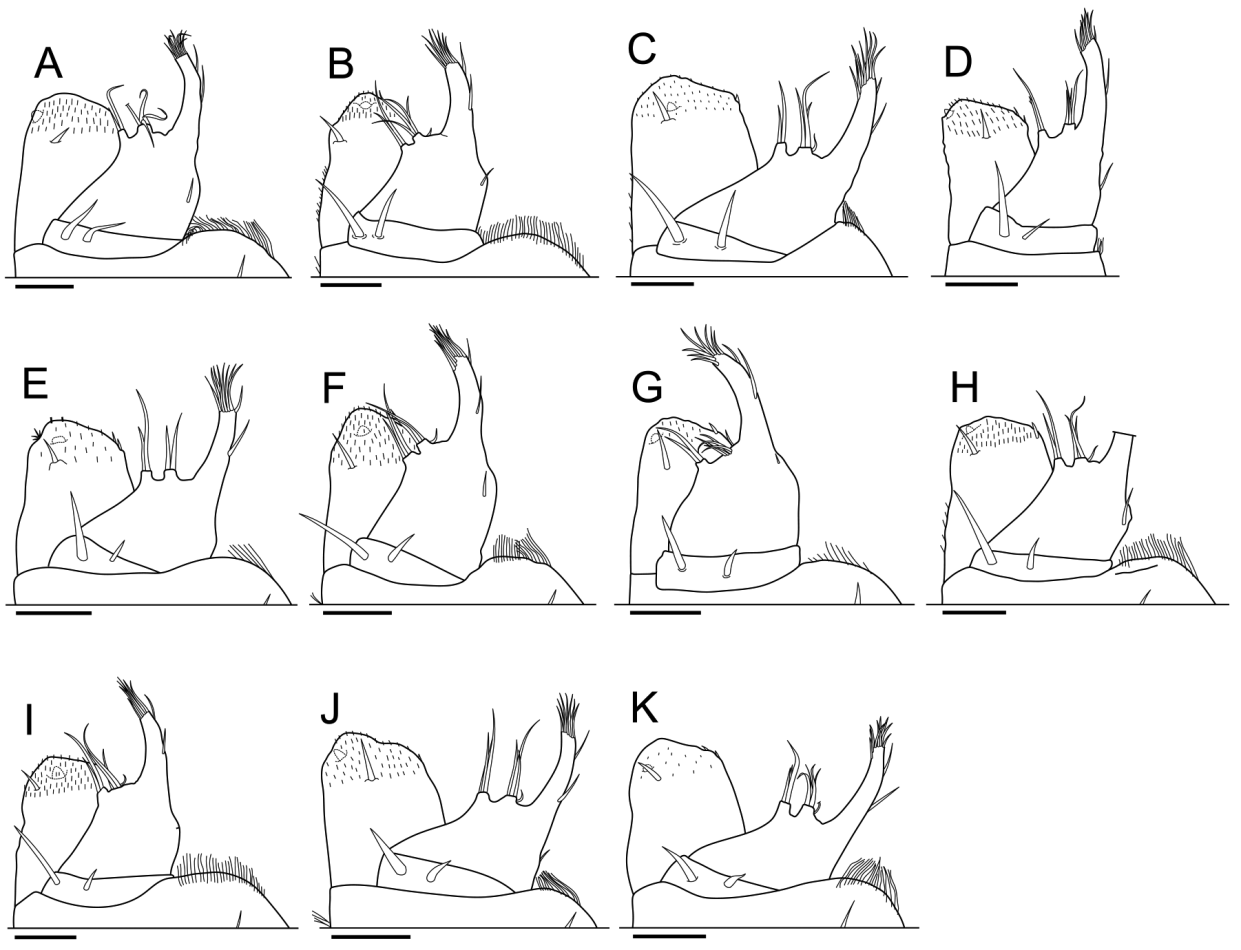
Maxillipeds. **A** *Burmoniscus aokii*, holotype **B** *Burmoniscus boninensis*, holotype **C** *Burmoniscus daitoensis*, holotype **D** *Burmoniscus hachijoensis*, holotype **E** *Burmoniscus japonicus*, holotype **F** *Burmoniscus kagoshimaensis*, holotype **G** *Burmoniscus murotoensis*, holotype **H** *Burmoniscus okinawaensis*, holotype **I** *Burmoniscus shibatai*, holotype **J** *Burmoniscus tanabensis*, holotype **K** *Burmoniscus watanabei*, Paratype (Cr-5350). All specimens male. Scale bars: 50 µm.

**Maxilliped.** This could be described on the basis of holotypes or paratypes (Fig. 5), although the apical part of the palp of the holotype of *Burmoniscus okinawaensis* was broken. The rectangular endite is covered by numerous minute setae at the distal margin, which also bears a small penicil and a stout spine. The palp consists of triangular apical and rectangular basal articles. The apical article bears a bundle of fine setae at the tip and two clumps of several long setae in the mid regions, and the basal article has one long and one short spine. There were some errors in the original descriptions of these features. For example, the original description of *Burmoniscus kagoshimaensis* does not show two groups of setae in the mid region of the apical article of the palp, whereas the holotype in fact bears them (Fig. 5). In the Remarks of the original description of *Burmoniscus hachijoensis*, the less numerous bifid setae on the maxilliped were used for species delimitation, but the presence of any bifid setae on the maxilliped could not be confirmed in the holotype nor in the figure by Nunomura (2007). Moreover, Nunomura (1986) argued that a bare endite is an important taxonomic character (e.g., in *Burmoniscus boninensis* and *Burmoniscus shibatai*), but present observations suggest that the apical margin of the endite of all species is covered by minute setae. Another erroneous omission can be mentioned. Taiti and Ferrara (1986) regarded that the penicil on the endite of the maxilliped as a defining taxonomic character of the genus *Burmoniscus*, but the original descriptions of the Japanese species did not mention such a penicil, which the present study has confirmed to be universally present (Fig. 5). Thus, the morphological characteristics of the maxilliped as originally described appear unsuitable for use as defining taxonomic characteristics for these species.

**Figure 6.**
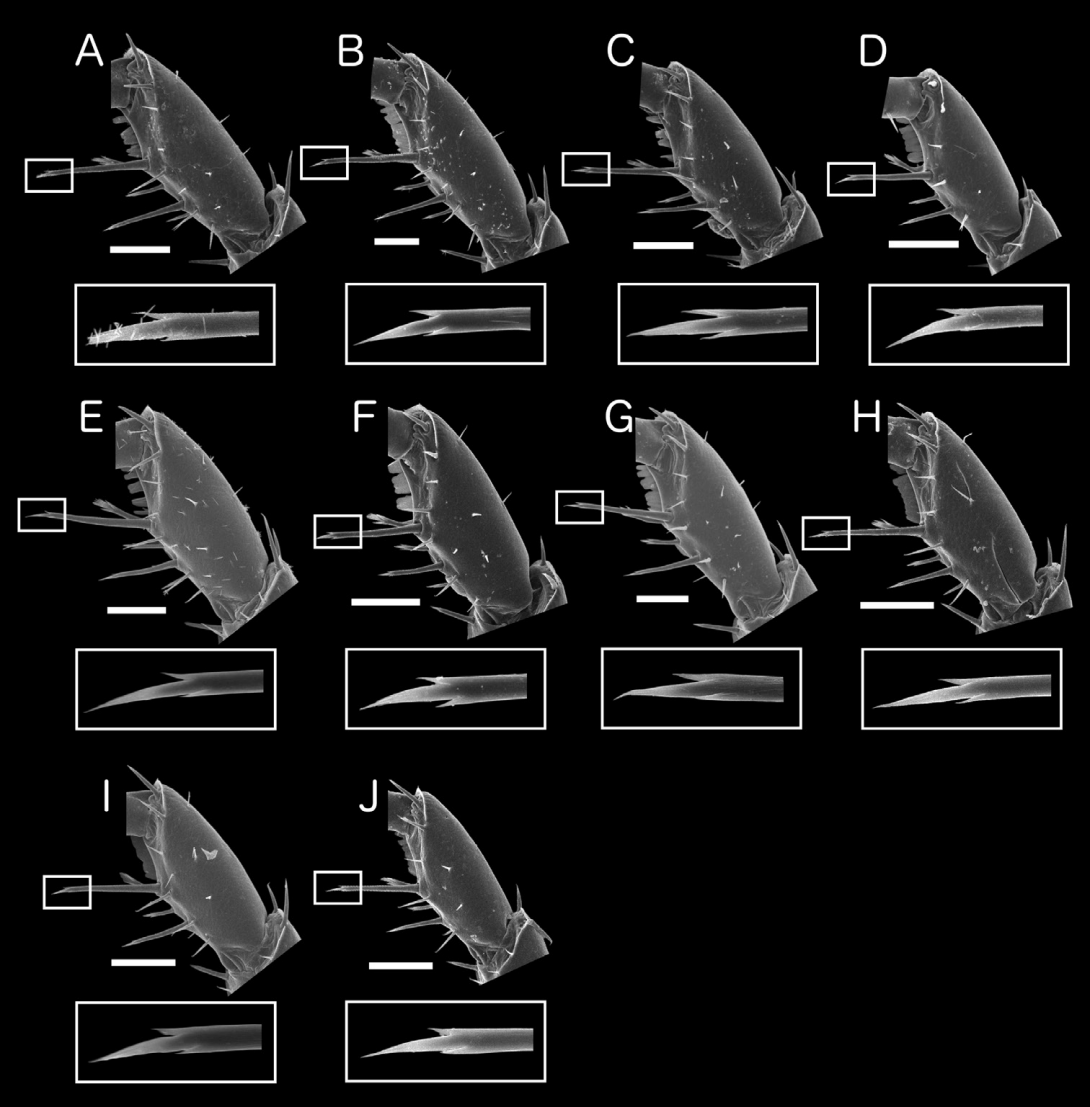
SEM photos of carpus of pereopod 1, with details of tip of longest seta. **A** specimen collected from Chichijima Island (including type localities of *Burmoniscus aokii* and *Burmoniscus boninensis*), KMNH-IvR-500810 **B** *Burmoniscus daitoensis*, KMNH-IvR-500811 **C** *Burmoniscus hachijoensis*, KMNH-IvR-500815 **D** *Burmoniscus japonicus*, KMNH-IvR-500818 **E** *Burmoniscus kagoshimaensis*, KMNH-IvR-500822 **F** *Burmoniscus murotoensis*, KMNH-IvR-500825 **G** *Burmoniscus okinawaensis*, KMNH-IvR-500831 **H** *Burmoniscus shibatai*, KMNH-IvR-500834 **I** *Burmoniscus tanabensis*, KMNH-IvR-500837 **J** *Burmoniscus watanabei*, KMNH-IvR-500842. All specimens male except A (female). Scale bars: 100 µm.

**Carpus of pereopod 1.** Nunomura (1986) did not describe the fine characteristics of the longest seta on the inner margin of the carpus of pereopod 1. Subsequently, however, this trait was used to distinguish *Burmoniscus kagoshimaensis* and *Burmoniscus tanabensis* (Nunomura 2003a,b), although there were inconsistencies in the descriptions. Nunomura (2003b) described the long seta of the carpus of *Burmoniscus kagoshimaensis* as being bifurcate in the Description, but in the Remarks he considered the absence of a bifid seta on pereopod 1 to be one of the defining taxonomic characters for *Burmoniscus kagoshimaensis*, based on comparison with *Burmoniscus okinawaensis*. Nunomura (2003a) also noted that *Burmoniscus tanabensis* had a simple long seta on the carpus and argued that the simple seta was an important taxonomic characteristic of this species. SEM photos of the carpus of pereopod 1 were obtained in the present study from topotypic specimens (Fig. 6). The longest seta was located on the middle of the inner margin of the carpus and the second longest seta was located in the basal region. The SEM photos revealed that the tip of the longest setae of all species is trifurcate, although the branches are very small and often difficult to observe. These observations suggest that the descriptions of this seta by Nunomura (2003a,b) were erroneous. Moreover, SEM photos revealed that the morphological features of the carpus of all species are consistent with those of *Burmoniscus kathmandius* as described by Schmalfuss (1983, fig. 22).

**Figure 7.**
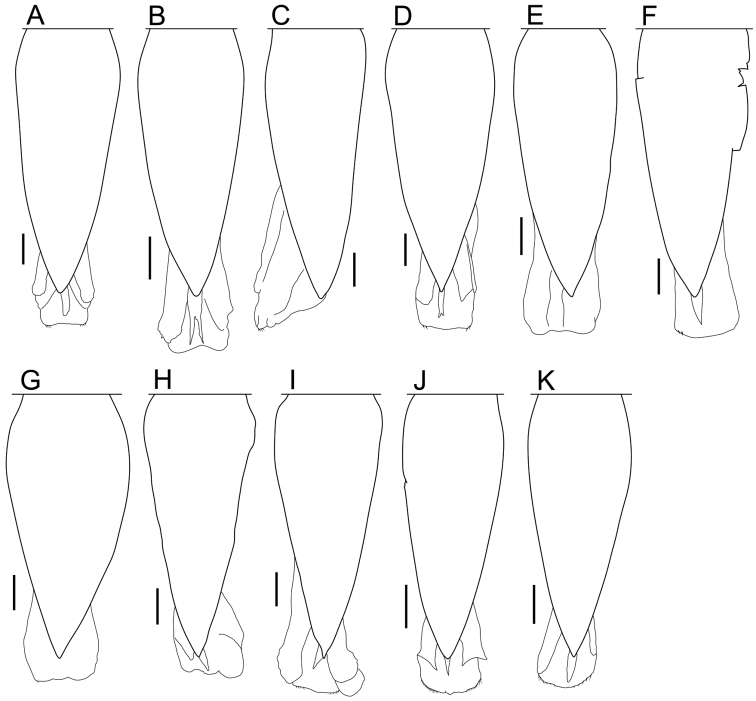
Genital papilla. **A** *Burmoniscus aokii*, holotype **B** *Burmoniscus boninensis*, paratype (Cr-5506) **C** *Burmoniscus daitoensis*, holotype **D** *Burmoniscus hachijoensis*, KMNH-IvR-500816 **E** *Burmoniscus japonicus*, KMNH-IvR-500819 **F** *Burmoniscus kagoshimaensis*, holotype **G** *Burmoniscus murotoensis*, holotype **H** *Burmoniscus okinawaensis*, KMNH-IvR-500832 **I** *Burmoniscus shibatai*, holotype **J** *Burmoniscus tanabensis*, holotype **K** *Burmoniscus watanabei*, paratype (Cr-5350). All specimens male. Scale bars: 50 µm.

**Genital papilla.** The morphological characteristics of the genital papilla of terrestrial isopods typically exhibit little variation among related species. In contrast, the original descriptions of the Japanese species of *Burmoniscus* (Nunomura 1986, 2003a, b, 2007) suggested that the genital papillae could be separated into two types: 1) pointed at the tip, and 2) round or truncate. The former type was reportedly found in *Burmoniscus aokii*, *Burmoniscus daitoensis*, *Burmoniscus japonicus*, *Burmoniscus kagoshimaensis*, *Burmoniscus okinawaensis*, *Burmoniscus tanabensis*, and *Burmoniscus watanabei*, and the latter in *Burmoniscus boninensis*, *Burmoniscus hachijoensis*, *Burmoniscus murotoensis*, and *Burmoniscus shibatai* (Suppl. material 3). However, the present data suggest that these descriptions were incorrect. In all the examined species (Fig. 7), the genital papillae consist of a rectangular lobe at the tip and a ventral shield with a thickened cuticle. The shields of all species are morphologically similar and fusiform. The pointed type of papillae may represent the ventral shield only, whereas the round or truncate type may represent a ventral shield with a lobe. Thus, this morphological character is not reliable for use as a defining taxonomic character.

**Figure 8.**
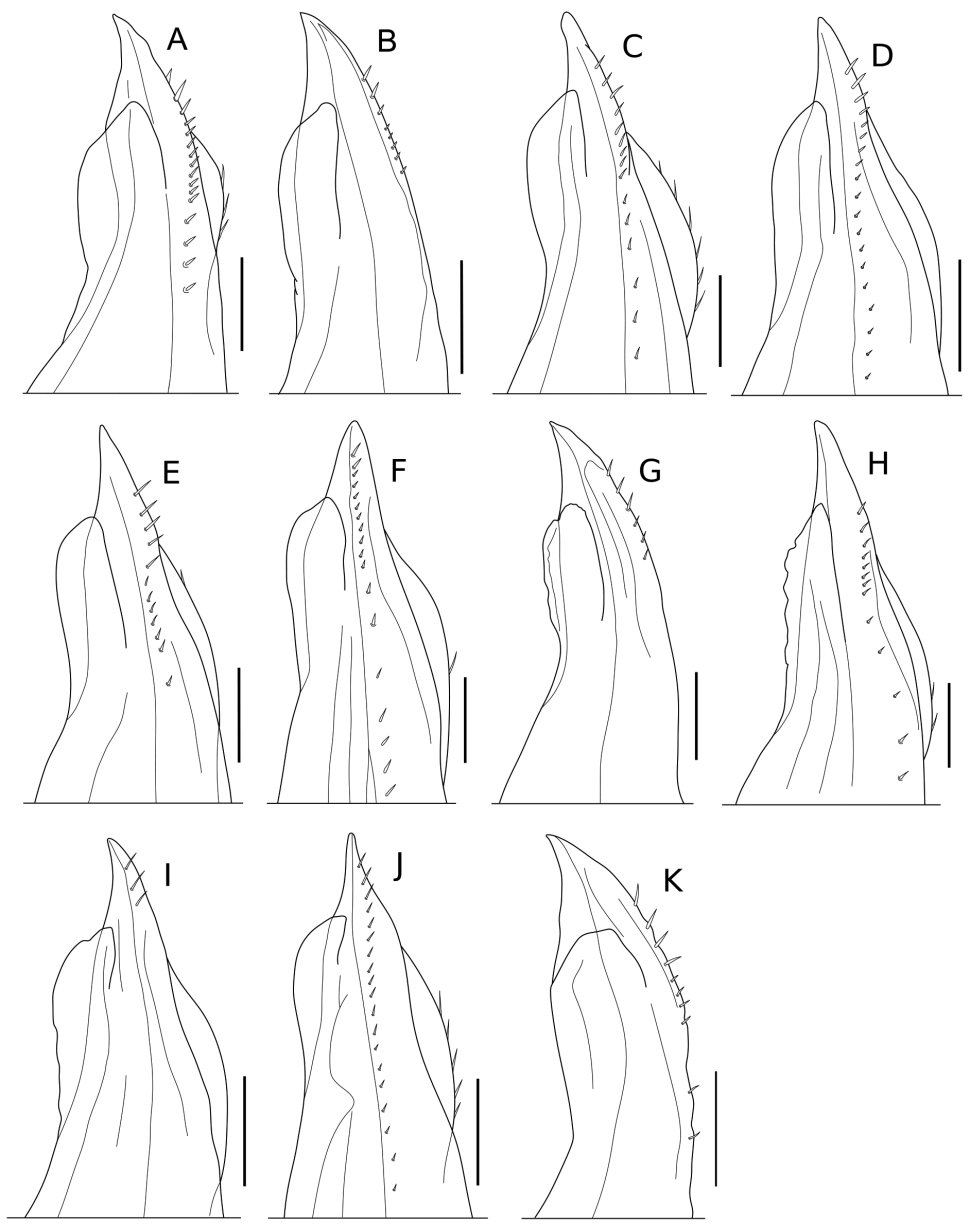
Pleopod 1 endopodite. **A** *Burmoniscus aokii*, holotype **B** *Burmoniscus boninensis*, paratype (Cr-5506) **C** *Burmoniscus daitoensis*, holotype **D** *Burmoniscus hachijoensis*, holotype **E** *Burmoniscus japonicus*, holotype **F** *Burmoniscus kagoshimaensis*, holotype **G** *Burmoniscus murotoensis*, holotype **H** *Burmoniscus okinawaensis*, KMNH-IvR-500832 **I** *Burmoniscus shibatai*, holotype **J** *Burmoniscus tanabensis*, holotype **K** *Burmoniscus watanabei*, paratype (Cr-5350). All specimens male. Scale bars: 50 µm.

**Figure 9.**
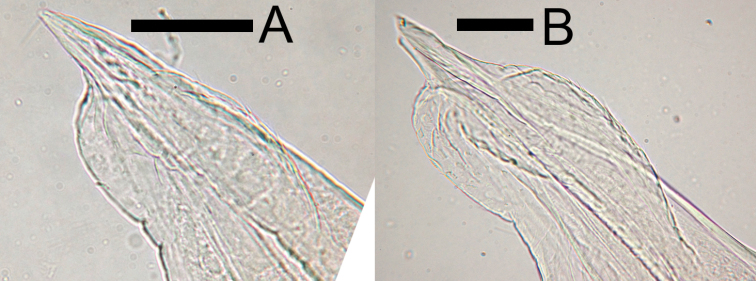
Photos of pleopod 1 endopodite of two specimens (**A** and **B**) collected from type locality of *Burmoniscus okinawaensis* (personal collection). Both specimens male. Scale bars: 50 µm.

**Male pleopod 1 endopodite.** The morphological characteristics of the tip of the endopodite of pleopod 1 have often been used as defining taxonomic characteristics for males of the genus *Burmoniscus* (e.g., Taiti and Ferrara 1986; Kwon and Jeon 1993). *Burmoniscus kagoshimaensis* has two flap-like type structures at the tip, and Nunomura (2003b) pointed out that this structure differs from those of *Burmoniscus okinawaensis*. The present reexamination of the holotype of *Burmoniscus kagoshimaensis* has shown, however, that they are indeed similar to those of *Burmoniscus okinawaensis* (Fig. 8). Moreover, the original descriptions suggested that several species have no or just one lobe-like structure at the tip, a characteristic that has been considered important for distinguishing species (Suppl. material 3). However, the present observations suggest that most species have a lobe-like structure on each side of the tip (Fig. 8). Exceptions are the paratypes of *Burmoniscus boninensis* and *Burmoniscus watanabei*, which have a lobe only on the outer margin, thus more or less consistent with Nunomura (1986). In sum, I conclude that any variation in the tip of this endopodite is no more than intraspecific variation, similar to that observed among specimens from the type locality of *Burmoniscus okinawaensis* (Fig. 9). Possession of a lobe on each margin at the tip is also characteristic of *Burmoniscus kathmandius* (fig. 23 in Schmalfuss 1983).

**Figure 10.**
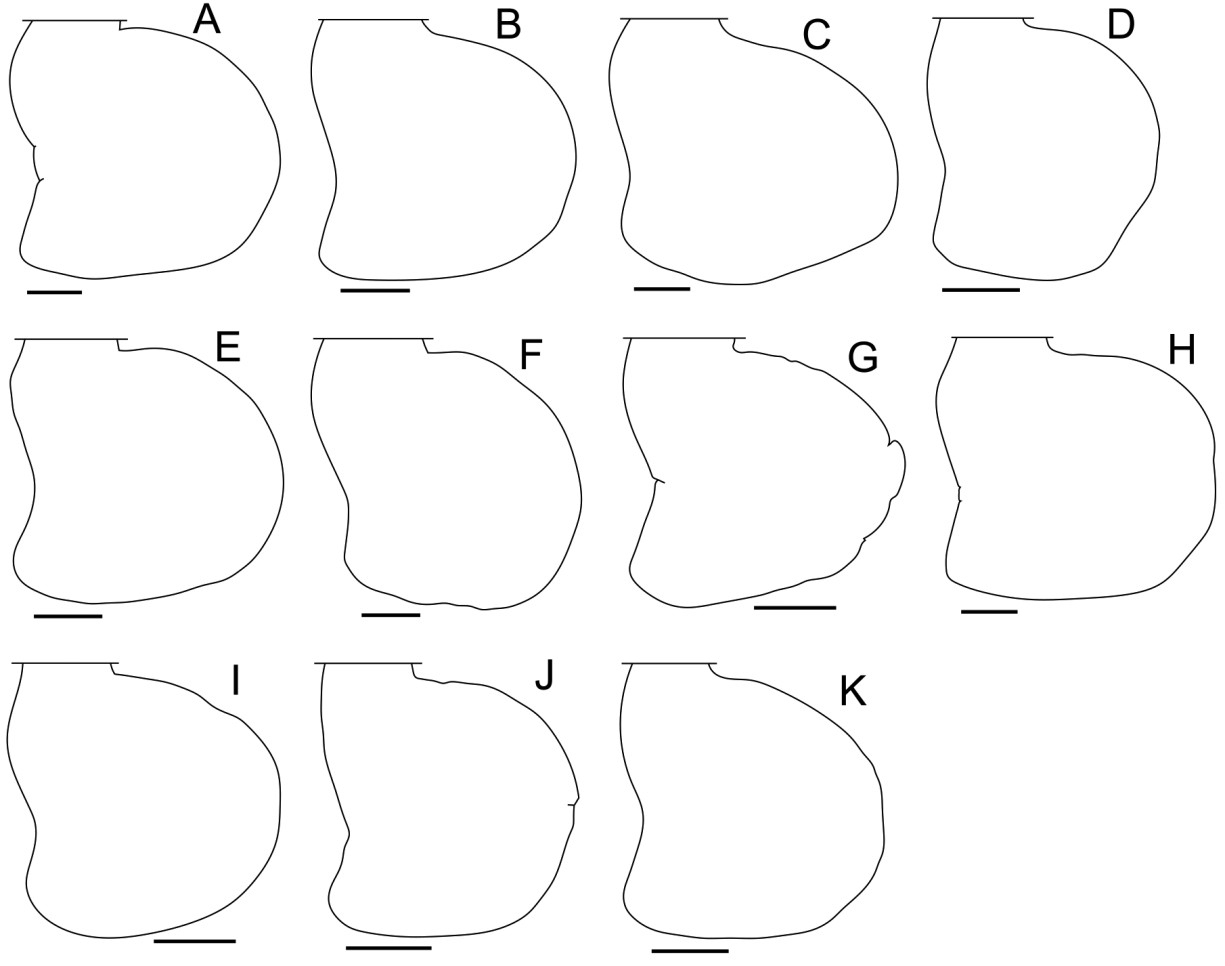
Pleopod 1 exopodite. **A** *Burmoniscus aokii*, holotype **B** *Burmoniscus boninensis*, paratype (Cr-5506) **C** *Burmoniscus daitoensis*, holotype **D** *Burmoniscus hachijoensis*, holotype **E** *Burmoniscus japonicus*, KMNH-IvR-500819 **F** *Burmoniscus kagoshimaensis*, KMNH-IvR-500821 **G** *Burmoniscus murotoensis*, holotype **H** *Burmoniscus okinawaensis*, KMNH-IvR-500832 **I** *Burmoniscus shibatai*, KMNH-IvR-500835 **J** *Burmoniscus tanabensis*, holotype **K** *Burmoniscus watanabei*, paratype (Cr-5350). All specimens male. Scale bars: 100 µm.

**Male pleopod 1 exopodite.** As with the endopodite, the morphological features of the exopodite of pleopod 1 in males are also important for the taxonomic differentiation of species of *Burmoniscus* (e.g., Schmalfuss 1983). In the original descriptions of the Japanese species, roughly three types of exopodite were recognized: semicircular, triangular, and rounded (Suppl. material 3). The present reexamination has revealed that all species have an exopodite with a shallow concavity on the outer margin and a rounded inner margin, although there are small morphological variations among the nominal species (Fig. 10). For example, *Burmoniscus hachijoensis* has a narrower exopodite, while that of *Burmoniscus daitoensis* and *Burmoniscus murotoensis* is wider than those of other species. However, this variation may be a function of specimen condition and/or growth. Moreover, the shape of the exopodite of the Japanese species is consistent with that of *Burmoniscus kathmandius* (fig. 5 in Schmalfuss 1983).

**Figure 11.**
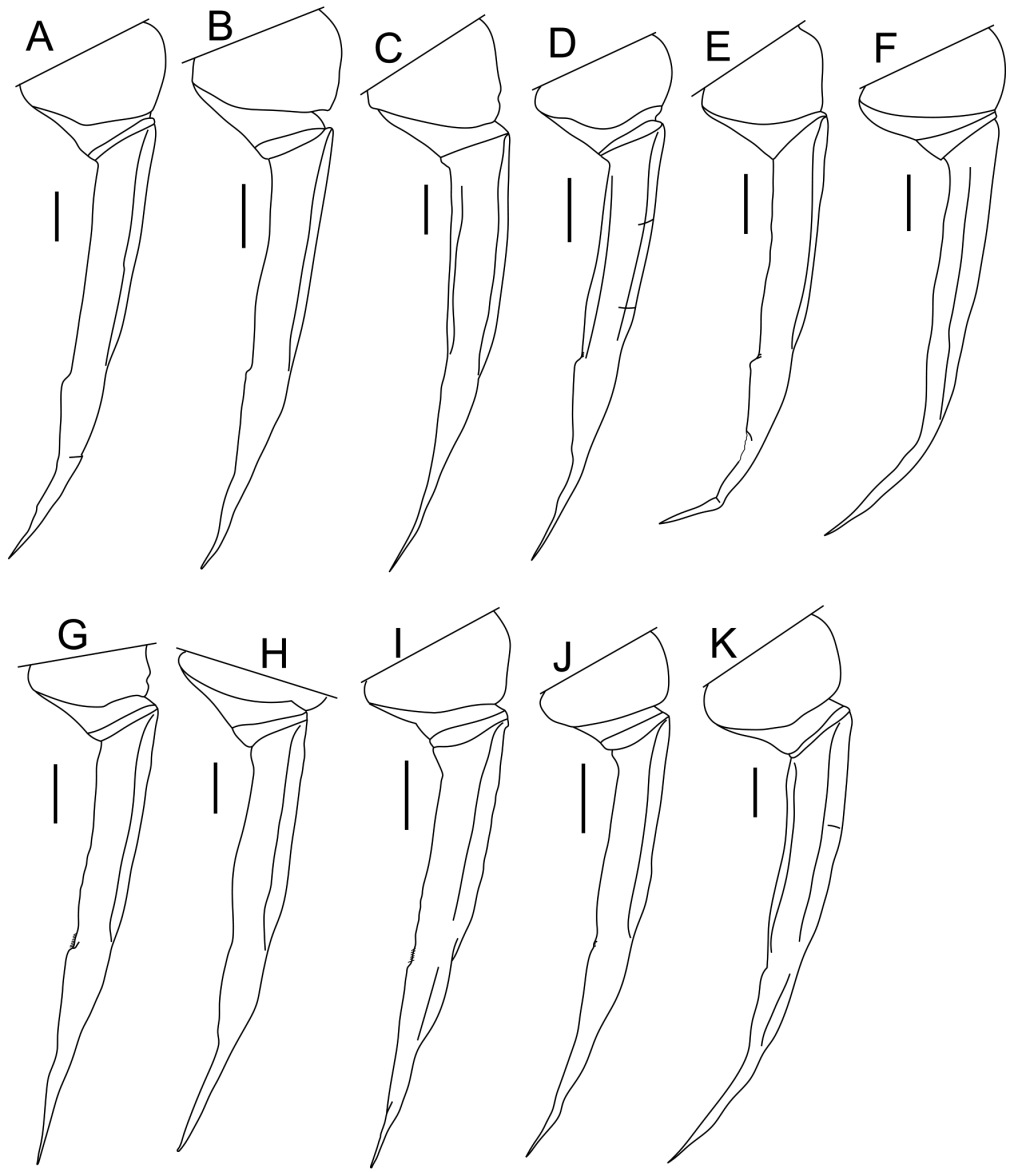
Pleopod 2 endopodite. **A** *Burmoniscus aokii*, holotype **B** *Burmoniscus boninensis*, paratype (Cr-5506) **C** *Burmoniscus daitoensis*, KMNH-IvR-500812 **D** *Burmoniscus hachijoensis*, holotype **E** *Burmoniscus japonicus*, holotype **F** *Burmoniscus kagoshimaensis*, holotype **G** *Burmoniscus murotoensis*, KMNH-IvR-500826 **H** *Burmoniscus okinawaensis*, KMNH-IvR-500832 **I** *Burmoniscus shibatai*, KMNH-IvR-500835 **J** *Burmoniscus tanabensis*, holotype **K** *Burmoniscus watanabei*, holotype. All specimens male. Scale bars: 100 µm.

**Male pleopod 2 endopodite.** The present reexamination of Japanese *Burmoniscus* has shown that the endopodite of male pleopod 2 of all the nominal species tapers towards the tip, although the extent of the curve at the tip varies among species (Fig. 11). The endopodites of *Burmoniscus japonicus*, *Burmoniscus kagoshimaensis*, and *Burmoniscus watanabei* have a greater outward curvature than those of the other species, but it is unclear whether such variation is useful for taxonomic differentiation of species. Nunomura (1986) used the form of the endopodite as a defining taxonomic trait for *Burmoniscus japonicus*, *Burmoniscus daitoensis*, *Burmoniscus boninensis*, and *Burmoniscus aokii*. However, the lengths of the endopodite of *Burmoniscus japonicus* and *Burmoniscus boninensis* were almost equal to those of other species. Moreover, Nunomura (1986) concluded that the shape of both lobes of pleopod 2 was an important character for identifying *Burmoniscus daitoensis* and *Burmoniscus aokii*, but his figures and my observations suggested that neither the endopodite nor the exopodite (see below) of pleopod 2 has two lobes. Taken together, these observations suggest that the endopodite of male pleopod 2 is unsuitable for differentiating among species of *Burmoniscus*.

**Figure 12.**
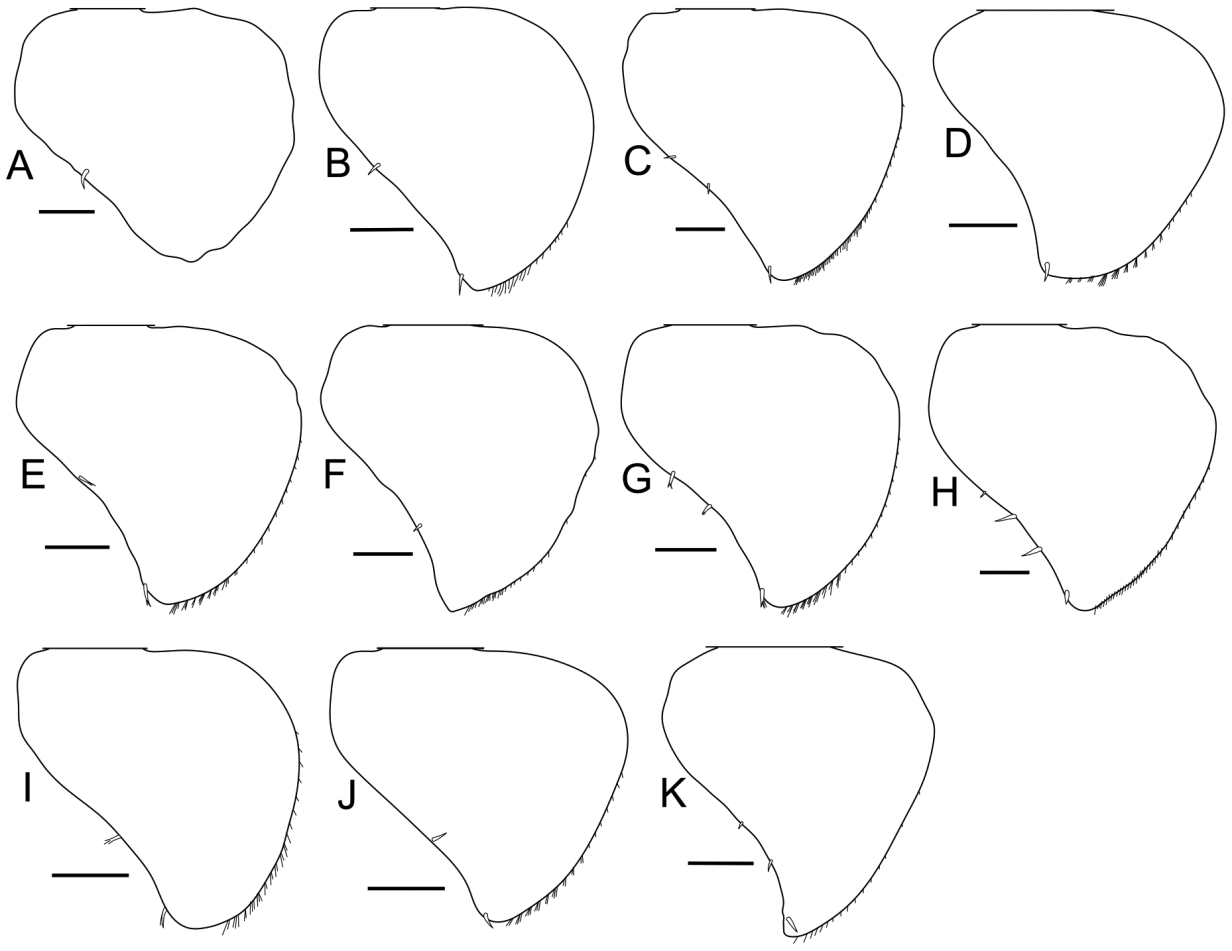
Pleopod 2 exopodite. **A** *Burmoniscus aokii*, holotype **B** *Burmoniscus boninensis*, paratype (Cr-5506) **C** *Burmoniscus daitoensis*, KMNH-IvR-500812 **D** *Burmoniscus hachijoensis*, holotype **E** *Burmoniscus japonicus*, KMNH-IvR-500819 **F** *Burmoniscus kagoshimaensis*, holotype **G** *Burmoniscus murotoensis*, KMNH-IvR-500826 **H** *Burmoniscus okinawaensis*, KMNH-IvR-500832 **I** *Burmoniscus shibatai*, KMNH-IvR-500835 **J** *Burmoniscus tanabensis*, holotype **K** *Burmoniscus watanabei*, paratype (Cr-5350). All specimens male. Scale bars: 100 µm.

**Male pleopod 2 exopodite.** Depending on species, the exopodite of pleopod 2 has been described as semicircular, triangular, or round in the original descriptions (Suppl. material 3), but these differences have not been used to distinguish among the species of *Burmoniscus* (Nunomura 1986, 2003a,b, 2007). The present reexamination has shown that all the exopodites are actually very similar, i.e. triangular with a rounded inner margin (Fig. 12).

**Figure 13.**
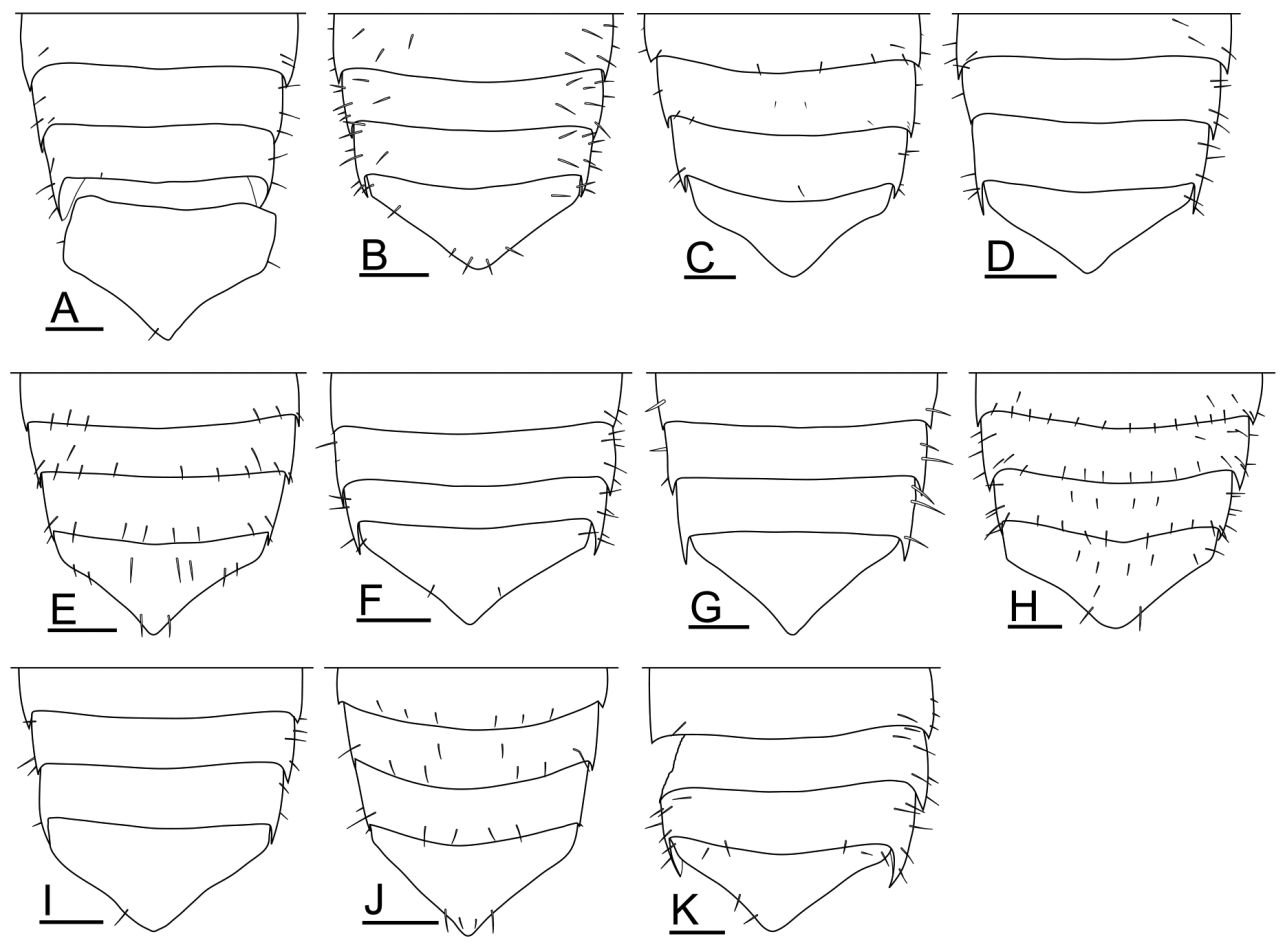
Pleonites 3–5 and pleotelson. **A** *Burmoniscus aokii*, holotype **B** *Burmoniscus boninensis*, paratype (Cr-5506) **C** *Burmoniscus daitoensis*, KMNH-IvR-500812 **D** *Burmoniscus hachijoensis*, holotype **E** *Burmoniscus japonicus*, KMNH-IvR-500819 **F** *Burmoniscus kagoshimaensis*, holotype **G** *Burmoniscus murotoensis*, holotype **H** *Burmoniscus okinawaensis*, KMNH-IvR-500832 **I** *Burmoniscus shibatai*, holotype **J** *Burmoniscus tanabensis*, KMNH-IvR-500840 **K** *Burmoniscus watanabei*, holotype. All specimens male. Scale bars: 100 µm.

**Pleon and pleotelson.** The length of the pleon and the shape of the posterior part of the pleotelson were previously used as distinguishing taxonomic characteristics for *Burmoniscus japonicus* and *Burmoniscus murotoensis*, respectively (Nunomura 1986). The present reexamination revealed no difference in the lengths and widths of any pleonite among all species (Fig. 13). The shape of the posterior section of the pleotelson exhibits some variation, however, e.g., tapering versus rounded (Fig. 13). Nunomura (1986) described the pleotelson of *Burmoniscus murotoensis* as being truncate posteriorly, but the holotype actually has a posteriorly tapered pleotelson. This discrepancy suggests that the taxonomic characters defined by Nunomura (1986) are not suitable for distinguishing among the two species. Instead, morphological variation in the posterior part of the pleotelson likely represents intraspecific variation, so cannot be used to distinguish among the Japanese species of *Burmoniscus*.

**Figure 14.**
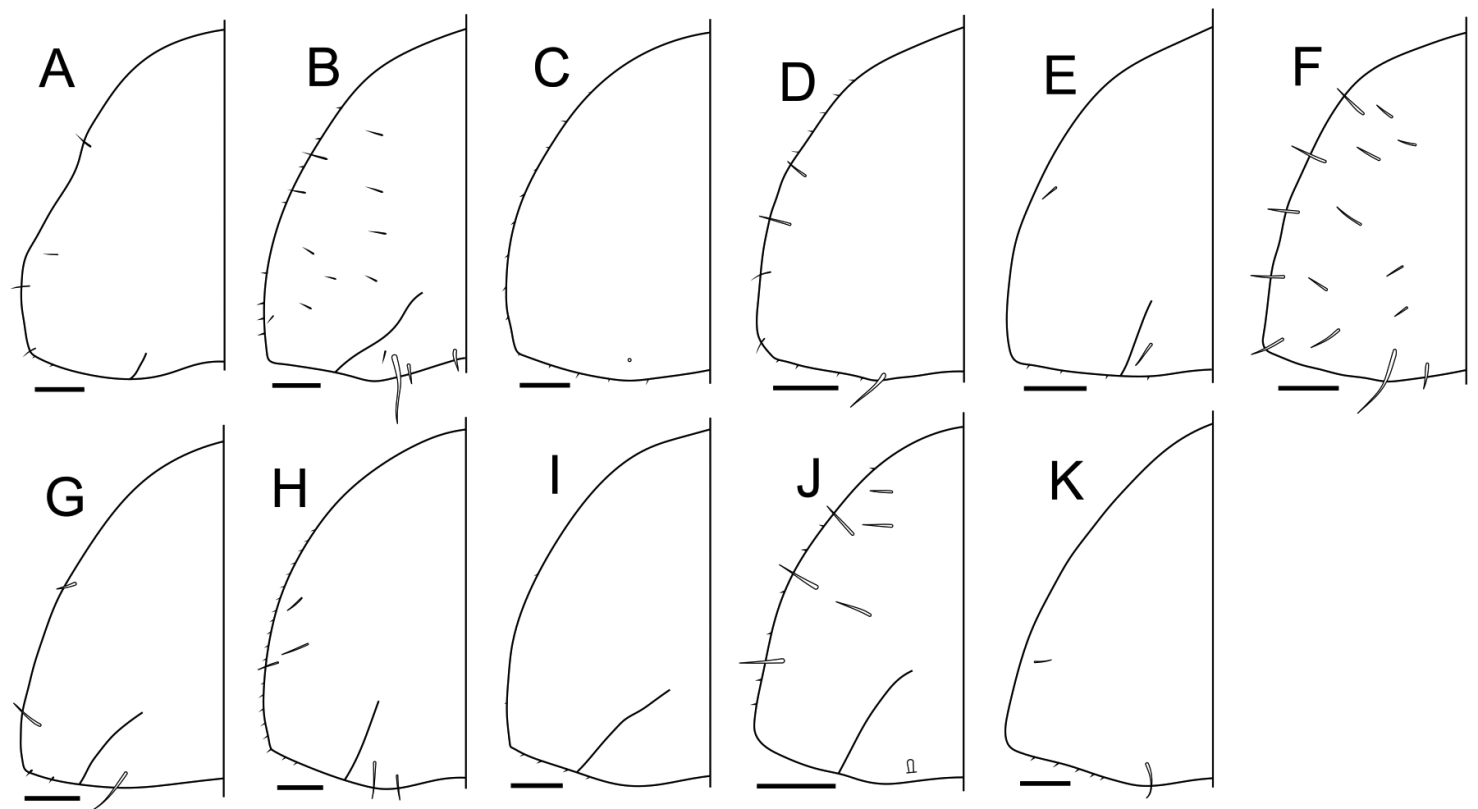
Epimera 7. **A** *Burmoniscus aokii*, holotype **B** *Burmoniscus boninensis*, holotype **C** *Burmoniscus daitoensis*, holotype **D** *Burmoniscus hachijoensis*, holotype **E** *Burmoniscus japonicus*, holotype **F** *Burmoniscus kagoshimaensis*, holotype **G** *Burmoniscus murotoensis*, holotype **H** *Burmoniscus okinawaensis*, KMNH-IvR-500832 **I** *Burmoniscus shibatai*, holotype **J** *Burmoniscus tanabensis*, holotype **K** *Burmoniscus watanabei*, holotype. All specimens male. Scale bars: 100 µm.

**Epimera 7.** The original descriptions did not describe epimera 7 explicitly (Suppl. material 3). However, in the Remarks for *Burmoniscus kagoshimaensis* it was cited in vague terms, “shape of postero-lateral margin of pereonal somite 7”, as a feature distinguishing this from *Burmoniscus okinawaensis* (Nunomura 2003b). The present reexamination of the postero-lateral margin of epimeron 7 showed no difference in shape between *Burmoniscus kagoshimaensis* and *Burmoniscus okinawaensis* (Fig. 14).

**Figure 15.**
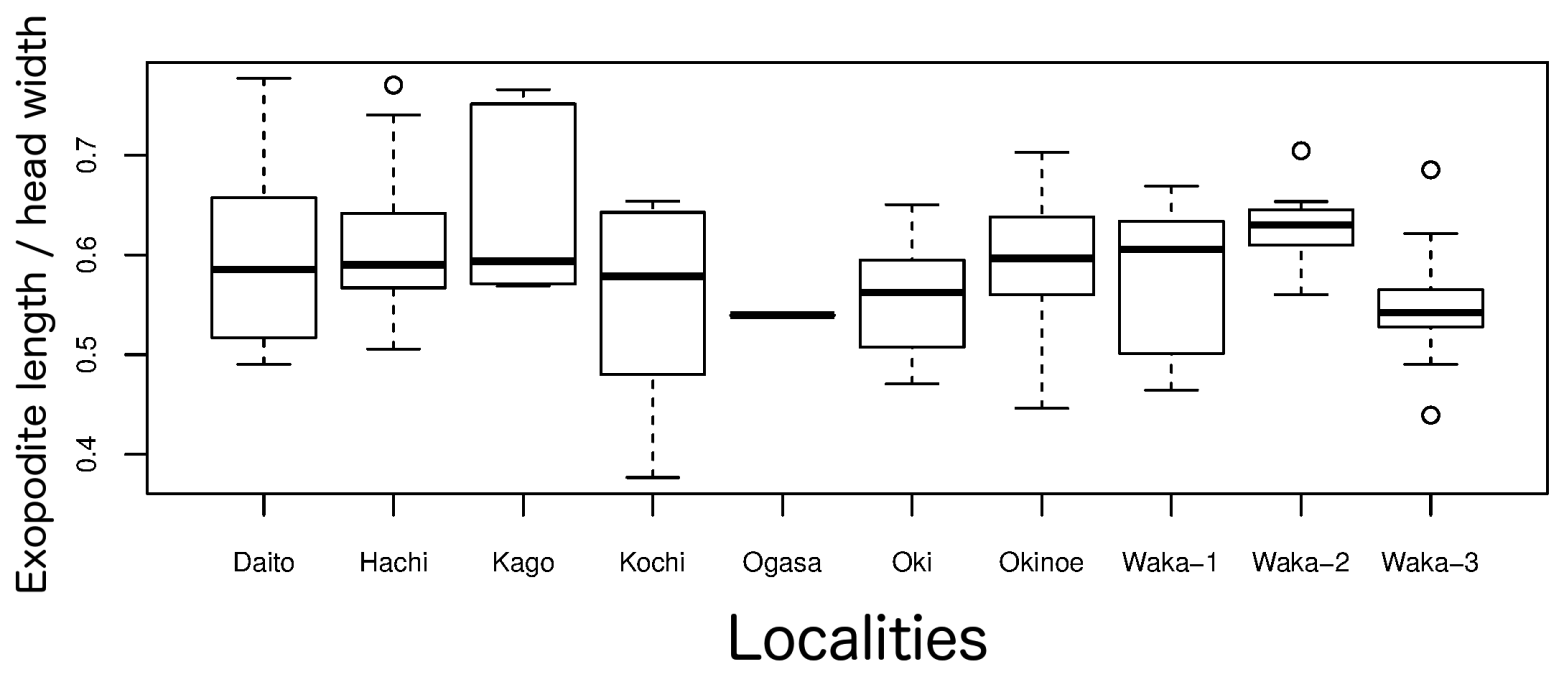
Median values and ranges of proportional length of uropodal exopodite, with respect to width of head, of *Burmoniscus* samples collected from type localities. The names of sampling sites are given in Figure 1. The lower and upper edges of each box mark the 25% and 75% percentiles. The whiskers represent the largest and smallest observed values, except for existing outliers. All specimens male.

**Uropods.** Uropods vary in length and have been used as a taxonomic characteristic to distinguish among some species. Original descriptions have often compared the length of the endopodite and exopodite (Suppl. material 3). The uropodal exopodites of 99 new specimens collected from the respective type localities were measured and compared using the ratio of exopodite length to head width among sites to avoid the confounding effect of body size (Fig. 15). The median value at each site ranged from 0.54 to 0.63; there was considerable variation within a site, and the ranges overlapped among the sites. Thus, the variation is too considerable for this feature to be useful in taxonomy.

**Figure 16.**
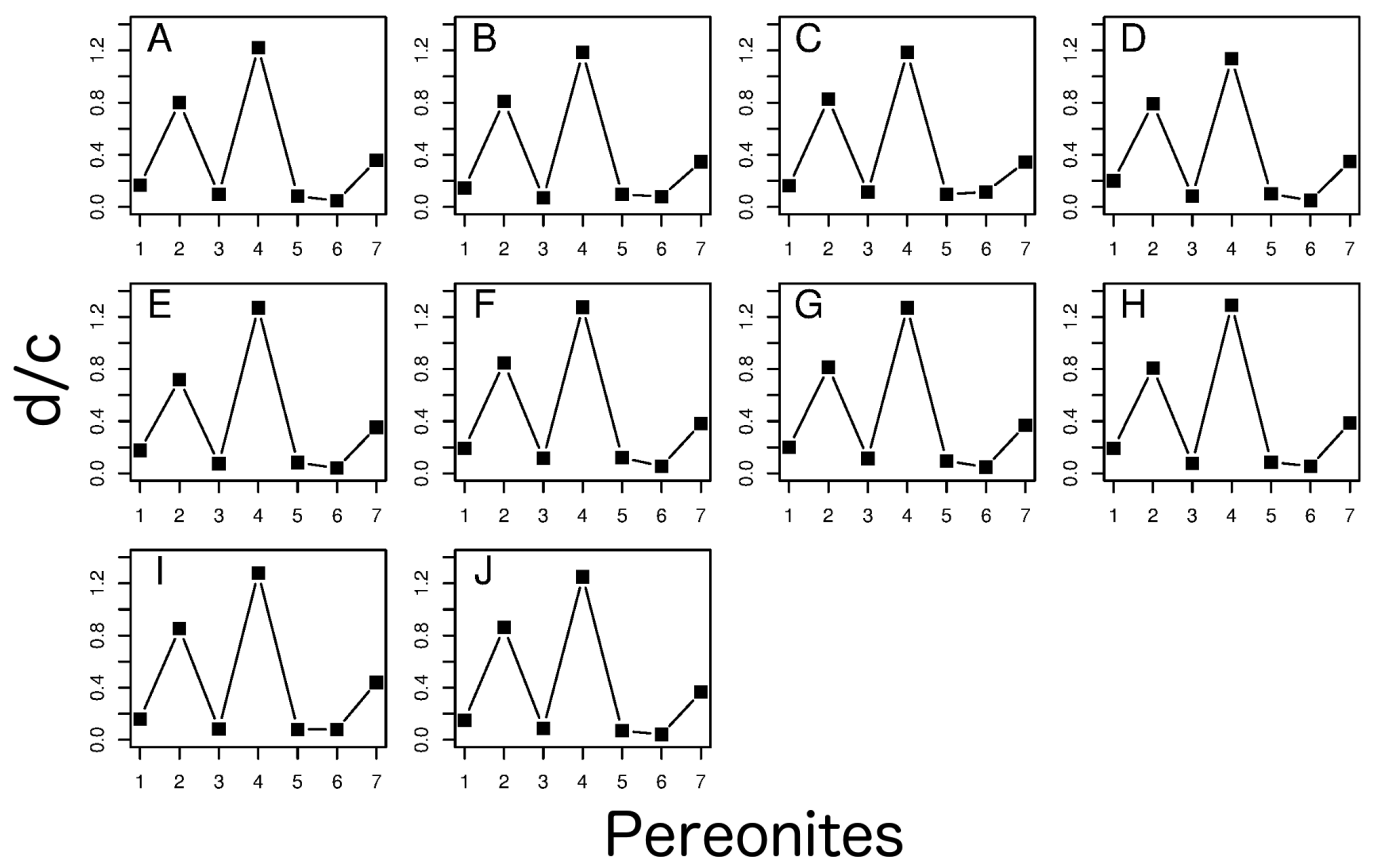
The d/c co-ordinate values of the *noduli laterales*. **A** specimens collected from Chichijima Island (including type localities of *Burmoniscus aokii* and *Burmoniscus boninensis*), KMNH-IvR-500809 **B** *Burmoniscus daitoensis*, KMNH-IvR-500813 **C** *Burmoniscus hachijoensis*, KMNH-IvR-500816 **D** *Burmoniscus japonicus*, KMNH-IvR-500820 **E** *Burmoniscus kagoshimaensis*, KMNH-IvR-500823 **F** *Burmoniscus murotoensis*, KMNH-IvR-500827 **G** *Burmoniscus okinawaensis*, KMNH-IvR-500833 **H** *Burmoniscus shibatai*, KMNH-IvR-500836 **I** *Burmoniscus tanabensis*, KMNH-IvR-500841 **J** *Burmoniscus watanabei*, KMNH-IvR-500843. All specimens male.

**Figure 17.**
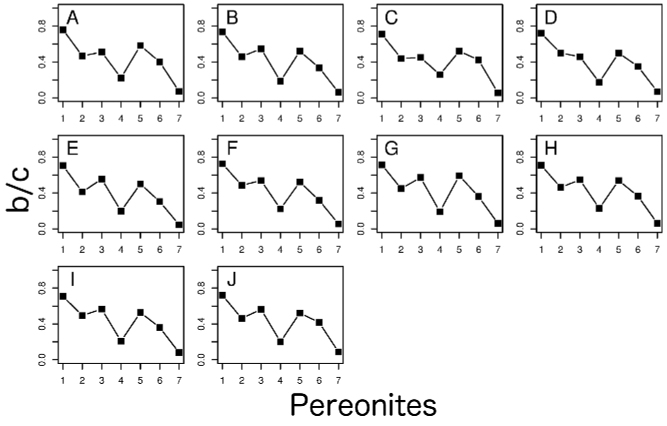
The b/c co-ordinate values of the *noduli laterales*. **A** specimens collected from Chichijima Island (including type localities of *Burmoniscus aokii* and *Burmoniscus boninensis*), KMNH-IvR-500809 **B** *Burmoniscus daitoensis*, KMNH-IvR-500813 **C** *Burmoniscus hachijoensis*, KMNH-IvR-500816 **D** *Burmoniscus japonicus*, KMNH-IvR-500820 **E** *Burmoniscus kagoshimaensis*, KMNH-IvR-500823 **F** *Burmoniscus murotoensis*, KMNH-IvR-500827 **G** *Burmoniscus okinawaensis*, KMNH-IvR-500833 **H** *Burmoniscus shibatai*, KMNH-IvR-500836 **I** *Burmoniscus tanabensis*, KMNH-IvR-500841 **J** *Burmoniscus watanabei*, KMNH-IvR-500843. All specimens male.

***Noduli laterales*.** In the original descriptions, the position of the *noduli laterales* was used as a taxonomic characteristic to distinguish among *Burmoniscus boninensis*, *Burmoniscus kagoshimaensis*, *Burmoniscus murotoensis*, *Burmoniscus okinawaensis*, *Burmoniscus shibatai*, and *Burmoniscus watanabei* (Nunomura 1986). Nunomura (1986) described variation in how far the *noduli laterales* on pereonite 2 extended from the lateral margin and concluded that the variation was sufficient to constitute a taxonomic difference. Moreover, Nunomura (2003b) argued that the remote position of the *noduli laterales* on pereonite 4 was an important characteristic for separating *Burmoniscus tanabensis* from *Burmoniscus okinawaensis* (Suppl. material 3). Taiti and Ferrara (1986) argued that the position of the *noduli laterales* is an important diagnostic characteristic for the genus *Burmoniscus*, but not of the species within it. They concluded that all species of this genus have one *nodulus lateralis* per side on each pereonite and the d/c co-ordinates exhibit clear peaks on pereonites 2 and 4. This contradicts most of the original descriptions. The present reexamination has shown that all species described by Nunomura (1986) have *noduli laterales* on pereonite 4 near the lateral margin (Suppl. material 3) and the newly calculated d/c and b/c co-ordinates reported herein for new specimens collected from type localities show that the setae on pereonites 2 and 4 are remote from the lateral margin in all species (Figs 16, 17). This pattern is identical to that of *Burmoniscus okinawaensis* collected from Hawaii (Taiti and Ferrara 1991) and also consistent with the genetic diagnosis of Taiti and Ferrara (1986).

**Figure 18.**
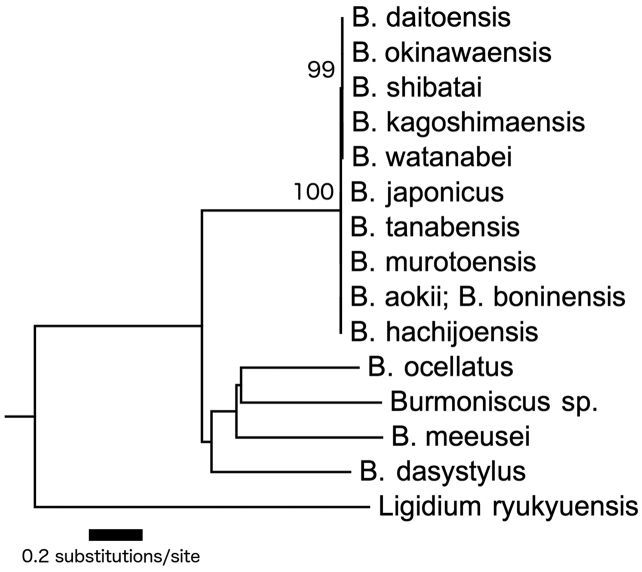
ML phylogenetic tree based on combined COI, 12S rRNA, and 16S rRNA sequence data. A specimen collected from a site on Chichijima Island was used in this analysis in lieu of specimens collected from the type localities of *Burmoniscus aokii* and *Burmoniscus boninensis*. Bootstrap values exceeding 90% are shown at each relevant node.

**Molecular analysis.** The total alignments of the three sequenced regions contained 1210–1243 bases. The 50% majority-rule consensus tree produced by the ML analysis is shown in Fig. 18. This analysis could not fully clarify the phylogenetic relationships among the 14 species of *Burmoniscus* in Japan, but four species, *Burmoniscus* sp., *Burmoniscus ocellatus*, *Burmoniscus dasystylus*, and *Burmoniscus meeusei*, exhibited distinct genetic independence from the others. The mean genetic difference (p-distance) among specimens collected from the type localities of eleven *Burmoniscus* species was 0.003, which is distinctively lower than what is usually regarded as interspecific-level differences in isopods (13–28% in Klossa-Kilia et al., 2006). The pairwise genetic distances among *Burmoniscus* sp., *Burmoniscus ocellatus*, *Burmoniscus dasystylus*, *Burmoniscus meeusei*, and grouped data of the other eleven *Burmoniscus* species ranged from 0.249 to 0.290, suggesting that these five, at least, are independent species. The present study also found two haplotypes in the eleven *Burmonicus* species. One of them was found in *Burmoniscus daitoensis*, *Burmoniscus kagoshimaensis*, *Burmoniscus okinawaensis*, *Burmoniscus shibatai*, and *Burmoniscus watanabei*, among which four species are distributed in southwestern Japan. The other haplotype was found in species distributed in eastern Japan. It may be interesting to investigate the history of gene flow and migration of these species, but clarifying this subject would require further analysis beyond the objectives of the present study.

## Conclusions

Based mostly on examination of type specimens and topotypic (or near-topotypic) material, I have re-described the morphological features and re-calculated various indices that were originally used for diagnosing and differentiating the eleven Japanese nominal species of *Burmoniscus*. They all exhibited little variation among species, and errors in some of the original description could be demonstrated. Based on these findings, it can be concluded that the species-level classification of Japanese *Burmoniscus* by Nunomura (1986, 2003a,b, 2007) is unsatisfactory, and instead it is proposed that there is a single species of *Burmoniscus* in Japan, as first proposed by Taiti and Ferrara (1991) and Kwon and Jeon (1993). Moreover, its morphological features are consistent with those of *Burmoniscus kathmandius*, so these eleven nominal species in Japan should be treated as junior synonyms of *Burmoniscus kathmandius*.

The present study has largely settled the taxonomic problems concerning *Brumoniscus* species in Japan, but one problem still remains unsolved. Nunomura (1986) compared some morphological characteristics of *Burmoniscus japaonicus* to those of *Setaphora truncata*, but the taxonomic status of this latter species is still doubtful. It was described by Dollfus (1898) on specimens from Indonesia (Celebes and Flores), but his description neglected some of the potentially diagnostic characteristics. Arcangeli (1927) recorded this species from Kyoto but it is not clear if these specimens are definitely conspecific with those from Indonesia. Clarifying the taxonomic status of *Setaphora truncata* and the relationship with *Burmoniscus kathmandius* requires observation of the holotype of the former, which I have not yet managed to locate. At the present stage of knowledge, it may be appropriate to treat *Setaphora truncata* and *Burmoniscus kathmandius* as different species. However, it is possible that the specimens of *Setaphora truncata* from Kyoto recorded by Arcangeli (1927) refer to *Burmoniscus kathmandius* but their reexamination is necessary to confirm this synonymy.

## Supplementary materials

Supplementary material 1 Type localities, collection data of new specimens, and accession numbers Data type: specimens data This dataset is made available under the Open Database License (http://opendatacommons.org/licenses/odbl/1.0/). The Open Database License (ODbL) is a license agreement intended to allow users to freely share, modify, and use this Dataset while maintaining this same freedom for others, provided that the original source and author(s) are credited.Shigenori Karasawa

Supplementary material 2 Details of specimens used for morphological observation Data type: specimens data This dataset is made available under the Open Database License (http://opendatacommons.org/licenses/odbl/1.0/). The Open Database License (ODbL) is a license agreement intended to allow users to freely share, modify, and use this Dataset while maintaining this same freedom for others, provided that the original source and author(s) are credited.Shigenori Karasawa

Supplementary material 3 Summary of diagnostic features of each nominal species of *Burmoniscus* in Japan according to the respective original descriptions Data type: measurement This dataset is made available under the Open Database License (http://opendatacommons.org/licenses/odbl/1.0/). The Open Database License (ODbL) is a license agreement intended to allow users to freely share, modify, and use this Dataset while maintaining this same freedom for others, provided that the original source and author(s) are credited.Shigenori Karasawa
